# A new era for intervention development studies

**DOI:** 10.1186/s40814-015-0032-0

**Published:** 2015-10-26

**Authors:** Pat Hoddinott

**Affiliations:** Chair in Primary Care, Nursing, Midwifery and Allied Health Professions Research Unit, Unit 13 Scion House, Stirling Innovation Park, Stirling, FK9 4NF UK

## Abstract

This editorial introduces a new special series on intervention development in the on-line open access journal *Pilot and Feasibility Studies.* An intervention development study reports the rationale, decision-making processes, methods and findings which occur between the idea or inception of an intervention until it is ready for formal feasibility, pilot or efficacy testing prior to a full trial or evaluation. This editorial begins to explore some of the challenges associated with this early research stage. It commences a debate about how to produce novel interventions which are fit for purpose and which solve important health and social care problems. By transparently reporting more intervention development studies, scientific rigour will be improved and everyone can learn from the experiences of others.

Intervention development can be viewed as a “black box” or the “Cinderella” of complex intervention trial design. This is because important processes and decision-making in the early stages of intervention development are seldom reported and until now, journals have shown little interest in publishing such studies. Intervention development studies exist in small grant reports and PhD chapters and tend to gather dust on the shelf as researchers move on to secure larger grants and new projects. So anecdotally, researchers encounter recurring pitfalls, spend time in blind alleys and worry about intervention decisions, with little guidance available. In addition, until recently, UK research funding institutions have not prioritised investment in complex intervention development.

The importance of methodological rigour at this early stage is recognised [1], and there is research waste from developing interventions that never impact on health care [2]. With ageing populations, multi-morbidity and lifestyle behaviours that seem remarkably resistant to change effective interventions are needed. In this special series, we begin to open the black box of intervention development. We are particularly interested in complex interventions, where there are several interacting components, rather than drugs or invasive devices which have regulated development processes.

## How are intervention development studies defined?

A working definition of an intervention development study for this special series is

*A study that describes the rationale, decision making processes, methods and findings which occur between the idea or inception of an intervention until it is ready for formal feasibility, pilot or efficacy testing prior to a full trial or evaluation.*

Put simply, it is a study about the what, why, how and when decisions involved in specifying an intervention, so that it can be replicated with fidelity by others. This is sometimes called an “intervention manual” which is prospectively written prior to a trial and includes any training required to deliver the intervention. This is a working definition of intervention development studies because there is remarkably little literature. Definitions and use of language are crucial. The UK National Institute of Health Research (NIHR) glossary does not define an intervention development study per se, but defines an intervention as

*The process of intervening on people, groups, entities or objects in an experimental study. In controlled trials, the word is sometimes used to describe the regimens in all comparison groups, including placebo and no-treatment arms* [3]*.*

It could be argued that the tail is wagging the dog (rather than the dog wagging its tail) in that intervention development studies are now likely to be shaped by the TIDieR guidance which aims to improve the systematic reporting of interventions [4].

There is a grey area around overlap with and definitions of feasibility studies. The NIHR definition of a feasibility study is “*Can this study be done?*” and expects that

*A robust case be made for the plausibility of the intervention and clinical importance of any subsequent full trial* [3].

Applying this definition, feasibility can apply within an intervention development study as described by Murray and colleagues in this issue [5] e.g. can these components work together? Are they likely to be summative, synergistic or detract? Feasibility can also apply to the operational aspects of trial design (e.g. recruitment, engagement, retention and outcome assessment) which do not feature in an intervention manual. The examples provided by Yardley and colleagues in this issue [6] make a strong case for assessing aspects of operational feasibility right from the start of intervention development. An extension of CONSORT guidelines for pilot and feasibility studies is in progress and this has given rise to much debate about the definitions of pilot and feasibility studies [7]. For example, the scope of the Medical Research Council (MRC) Public Health Intervention Development funding scheme [8] includes research to inform sample size, power calculations, and estimates of recruitment and retention which are usually assessed in a formal pilot or feasibility study.

## What do we know about intervention development studies?

Little is known about the number of intervention development studies that are tested in a formal pilot, proceed to a full evaluation and are implemented into routine practice. From 101 reports of new medical discoveries, over-optimism was evident as only one led to the development of an intervention that was widely used [9]. Optimistic bias is therefore important in intervention development studies. Assessing operational bias in systematic reviews is routine [10], and there are particular issues for assessing bias in behaviour change trials [11], but less attention has been paid to bias when developing an intervention.

What strategies might help to identify and reduce optimistic bias? Karl Popper proposed the discipline of falsification in the scientific method and the importance of challenging all knowledge, by proactively seeking to disconfirm it [12]. From thousands of observations of swans in the UK, you would conclude that all swans are white. Searching in unexpected places is required to find the black swan. Daniel Kahneman states overconfidence as endemic in medicine and describes the planning fallacy, where plans are unrealistically close to best case scenarios [13]. Kahneman recommends gaining an outside view, for example by examining statistical data from similar projects. Others highlight the importance of qualitative methods and triangulation approaches to critique or validate quantitative data and provide a personal perspective [14].

Groupthink [15] is where cognitively homogeneous groups have strong allegiances, tend not to voice dissent, rationalise away counterarguments and are confident in their plans. What happens within groups is complex [16], and strategies to avoid groupthink in the early stages of trial design have received little attention. Research teams and expert panels can benefit from selecting independent thinkers [17] to be the critical friend, the dragon in the den or devil’s advocate. Citation bias, where research teams with prestige are disproportionately cited, can be a problem which contributes to research waste [18]. Groupthink can result in premature conceptual closure, the collection and reporting of confirming data only and for “assumption habits” or blind spots to be unrecognised. A recent example is where a dominant behaviour change theory, the theory of planned behaviour, has underpinned interventions for three decades and is no longer considered valid [19]. It provides valuable insights into how un-expected findings can be attributed to operational flaws rather than questioning the validity of the theory itself. It can be argued, therefore, that methods which tend towards consensus (e.g. focus groups; Delphi techniques) are only appropriate when finalising an intervention specification.

Sampling, location and context bias should be considered, regardless of the methods used to develop an intervention. For example, when designing smartphone applications, sampling and location decisions can influence the data collected about the intervention use [20]. Certain groups or settings may not be included e.g. ethnic minorities, rural communities. Convenience sampling bias is where participants or settings overtly or covertly have something in common which limits generalizability. This may be unavoidable when resources are limited and can be accounted for in later pilot testing. Constructing a diversity sampling matrix [21] and using several interviewers from different disciplines to minimise interviewer bias are useful strategies to increase the range of perspectives. Proactively challenging and testing assumptions with follow-up of any counter-intuitive findings are important.

## Intervention development is seldom a fixed prospective linear process

It is apparent that isolating intervention development as a stand-alone research stage, study or report may not be appropriate. Even in pilot trials, defined as a “*smaller version of the main study used to test whether the components of the main study can all work together*” [3], further tinkering with the intervention may be warranted prior to a full trial. This is because health service contexts vary, change and interact with complex interventions in many different ways [22]. Unlike the constant enclosed environment of a petri dish for introducing new antibiotics into a microbe culture—interventions where human relationships form a significant part are dynamic and vulnerable to unexpected events. The meanings attributed to the intervention by people and the accompanying visuals, emotions and narrative are well recognised in commercial product development [5] but have been relatively neglected in health research. Innovation approaches are changing [23]. In the human computer interaction field, an intervention needs to constantly adapt to rapid innovation and a cyclical, iterative approach is recommended for generating hypotheses for mini tests [20, 24]. Mini tests or pilots generate data that can validate decisions and challenge critical assumptions at an earlier stage and quickly when developing an intervention [23]. This contrasts with more labour-intensive approaches where extensive modelling and theory generation precede any testing. Rapid validation mini tests can overcome one of the limitations of qualitative research in intervention development: when people are asked about hypothetical interventions, they can say what they find acceptable, need or want, but their subsequent actions can be quite different [23]. More tangible mini-testing processes within the intended intervention context have some parallels to action research [25] and quality improvement approaches like the plan-do-study-act cycle [26].

## What guidance is available?

The Medical Research Council guidance for complex interventions does not define an intervention development study per se but poses helpful questions for researchers [1]. In Table 1, I propose adaptations to the MRC Guidance questions to reflect more recent literature and debate.

**Table 1:** Questions for researchers about intervention development adapted from the MRC Complex Intervention guidance [1]

1. Are you clear about what you are trying to do, what outcome you are aiming for, and how you will bring about change?
2. Does your intervention have a coherent theoretical basis?
3. Have you used this theory systematically to develop the intervention?
4. Can you describe the intervention fully according to TIDieR guidelines [4], so that it can be implemented properly for the purposes of your evaluation and replicated by others?
5. Does the existing evidence [or lack of evidence]—ideally collated in a systematic review—support the development of your intervention so that it is likely to be feasible, effective and cost effective?
6. Has future implementation in multi-centre research settings and future translation into the real world been considered?
7. Has the potential for bias been considered e.g. optimistic bias, group think, sampling, location and context bias?

Questions 1–3 are unchanged. The TIDieR guidelines are incorporated into question 4. Question 5 is reworded to reflect the absence, heterogeneity or inadequate reporting of interventions in systematic reviews. Strategies for intervention synthesis to select the “best bets” when translating systematic review evidence into intervention development are proposed [27]. Novel intervention development may be justified, for example when a series of null trials in a particular context is counter to the international systematic review evidence [28], or existing interventions may have limited reach for particular groups, for example the uptake of weight loss interventions by men compared to women [29]. Question 6 is reworded to consider future implementation barriers and facilitators for both a full trial and translation into routine care. Question 7 is new and asks the following: has the potential for bias been considered? It proposes optimistic bias, group think, sampling, location and context biases as important examples.

There are many challenges ahead for intervention development studies. How do the academic community and policy makers weigh up the pros and cons of “slow research” versus efficient designs? How do research teams make the myriad of small decisions to finalise intervention manuals, particularly when systematic review evidence is lacking? How do traditional graded evidence [27], inductive, deductive and abductive logic [30], tacit and explicit knowledge [31], fast and slow thinking [13] and creativity contribute to intervention development? How can methods and decision-making processes be reported in a rigorous scientific and transparent way? How useful would checklists or frameworks be when commercial technology companies like Google (see https://research.google.com/) achieve rapid innovation with impact through flexibility?

## Moving forward

This series commences a debate which involves the research community, patients, clinicians, the public, charities and anyone with an interest in developing the best possible interventions for health and wellbeing. It aims to raise the profile and value of intervention development research, to open the black box and begin to unpack the experiences, methods, processes and outcomes. This will help to promote cross-disciplinary learning and stimulate debate about how to produce novel interventions to solve important health and social care problems. Intervention development is where creativity, science and art meet and the balance is delicate. Future qualitative synthesis of intervention development studies could have the potential to illuminate why early intervention promise so often disappears, resulting in research waste. Imperative are the principles of high-quality scientific reporting and rigour, with descriptions of how patients, the public, clinicians, relevant staff and policy makers are involved in the decision-making processes. Transparent reporting of both successes and failures is crucial.
